# Isolation of an Anti–tumour Disintegrin: Dabmaurin–1, a Peptide Lebein–1–like, from *Daboia mauritanica* Venom

**DOI:** 10.3390/toxins12020102

**Published:** 2020-02-05

**Authors:** Florence Chalier, Laura Mugnier, Marion Tarbe, Soioulata Aboudou, Claude Villard, Hervé Kovacic, Didier Gigmes, Pascal Mansuelle, Harold de Pomyers, José Luis, Kamel Mabrouk

**Affiliations:** 1 INP, Institut de Neurophysiopathologie, UMR 7051–CNRS & Aix–Marseille Université, Faculté de Pharmacie, 27 bd Jean Moulin, 13285 Marseille cedex 05, France; claude.villard@univ-amu.fr (C.V.); herve.kovacic@univ-amu.fr (H.K.); 2 ICR, Institut de Chimie Radicalaire, UMR 7273–CNRS & Aix–Marseille Université—Faculté des Sciences de Saint Jérôme, Avenue Escadrille Normandie Niémen, 13397 Marseille Cedex 20, France; laura.mugnier@hotmail.fr (L.M.); mariontarbe@hotmail.fr (M.T.); soioulata.ABOUDOU@univ-amu.fr (S.A.); didier.gigmes@univ-amu.fr (D.G.); 3 Latoxan Laboratory, Z.A Auréats, 845 Avenue Pierre Brossolette, 26800 PORTES lès VALENCE, France; harold.pomyers@latoxan.com; 4 IMM (MaP), Institut de Microbiologie de la Méditerranée (Marseille Protéomique), FR 3479—CNRS & Aix–Marseille Université, 31 Chemin Joseph Aiguier, 13009 Marseille, France

**Keywords:** angiogenesis, anti–angiogenic peptide, cell adhesion molecules, disintegrin, integrin, snake venom, endothelial cells, anti–tumour

## Abstract

In the soft treatment of cancer tumours, consequent downregulation of the malignant tissue angiogenesis constitutes an efficient way to stifle tumour development and metastasis spreading. As angiogenesis requires integrin–promoting endothelial cell adhesion, migration, and vessel tube formation, integrins represent potential targets of new therapeutic anti–angiogenic agents. Our work is a contribution to the research of such therapeutic disintegrins in animal venoms. We report isolation of one peptide, named Dabmaurin–1, from the hemotoxic venom of snake *Daboia mauritanica*, and we evaluate its potential anti–tumour activity through in vitro inhibition of the human vascular endothelial cell HMECs functions involved in tumour angiogenesis. Dabmaurin–1 altered, in a dose–dependent manner, without any significant cytotoxicity, HMEC proliferation, adhesion, and their mesenchymal migration onto various extracellular matrix proteins, as well as formation of capillary–tube mimics on Matrigel^TM^. Via experiments involving HMEC or specific cancers cells integrins, we demonstrated that the above Dabmaurin–1 effects are possibly due to some anti–integrin properties. Dabmaurin–1 was demonstrated to recognize a broad panel of prooncogenic integrins (αvβ6, αvβ3 or αvβ5) and/or particularly involved in control of angiogenesis (α5β1, α6β4, αvβ3 or αvβ5). Furthermore, mass spectrometry and partial N–terminal sequencing of this peptide revealed, it is close to Lebein–1, a known anti–β1 disintegrin from *Macrovipera lebetina* venom. Therefore, our results show that if Dabmaurin–1 exhibits in vitro apparent anti–angiogenic effects at concentrations lower than 30 nM, it is likely because it acts as an anti–tumour disintegrin.

## 1. Introduction

The venom toxins of *Daboia mauritanica,* a Viperidae snake found in north western Africa, target the haemostatic system. For victims, bites induce various first symptoms followed by haemorrhages and abnormalities in the blood’s coagulation system. The toxins involved in such profuse bleeding are metalloproteinases that could contain a disintegrin domain and are helped by simple disintegrin proteins, which interact with the integrin adhesion receptors [1]. A recent published fractionation work of the *Daboia mauritanica* venom components showed that several disintegrins encountered in other Macrovipera or Vipera venoms are contained by the Dm venom [2]. Integrins regulate cell–cell and cell–extracellular matrix contacts in physiology and disease. They allow cell adaptation to environmental changes, and cell anchorage, growth and motility, eliciting diverse signals for polarity, position and differentiation. Various integrins are involved in oncogenesis and cancer development [3]. Up or down regulation of expression of these integrins determine incidence of such diseases and the patient prognosis. The decrease of cell–matrix adhesion increases motility of cancer cells and stimulates cancer growth, invasion, and metastasis, whereas restoration of cell–matrix adhesion reverses this tendency [4]. On one hand, under–expressions of some integrins such as integrin α2β1 in colon carcinoma, have an anti–oncogenic effect [5]. On the other hand, over–expressions of others integrins such as α3β1 [6], αvβ or αvβ5 [7], or α6β4 [2], have a prooncogenic effect and are sometimes correlated with cancer aggressiveness. In addition, integrins α2β1 and α5β1 are involved in adhesion and differentiation, when integrins αvβ3, αvβ6, α6β4 are involved in proliferation and migration.

Furthermore, as integrins can also be overexpressed by the vascular endothelial cells recruited by tumour for formation of new blood capillaries [8], disintegrins may also present interesting anti–angiogenic effects [9,10]. In the absence of neo–angiogenesis, tumour is restricted to a microscopic size and tumour cells do not enter into circulation to initiate the process of metastasis [11]. Activation of angiogenesis, inducing tumour growth and metastasis escape, depends on proliferation, adhesion, migration, and maturation of the vascular endothelial cells recruited [12]. Several factors contribute to each individual process, and the development of new vessels is regulated by the balance between angiogenic activators and endogenous angiogenic inhibitors [7]. Expression of a integrin by endothelial cells (ECs) was guided by proangiogenic factors such as vascular endothelial or basic fibroblast or platelet–derived growth factors (VEGF, bFGF and PDGF respectively) secreted by tumour cells. Expression of integrin αvβ3was induced mainly by bFGF and mainly in blood neovessels [13]. Integrin αvβ3_,_ binds to vitronectin and fibronectin and allows EC migration and proliferation, but it binds also to matrix metalloproteinase 2 (MMP–2) which participates to degradation of extracellular matrix (ECM) in neoangiogenesis. Integrin α5β1 is stimulated by the same proangiogenic factor, but its ligand is only fibronectin. Expression of integrin αvβ5_,_ that targets vitronectin is induced by VEGF [14], and therefore, αvβ5 is surely involved in another step of angiogenesis. Disruption of the EC interactions, with the ECM proteins or with the endogenous MMP–2 [15] can inhibit angiogenesis. Most of the angiogenesis inhibitors in clinical trials for cancer treatment, target the EC integrins αvβ3 and αvβ5 [16]. Integrin α4β1 stimulated by both VEGF and bFGF has for ligands, fibronectin and the vascular cellular adhesion molecule (VCAM) which promotes the endothelial cell binding to muscle cells. Expressions of integrins α1β1 and α2β1 are stimulated by factors VEGF–A and –C. However, α1β1 gene silencing deactivates tumour angiogenesis and tumour development, when α2β1 gene silencing induces opposite actions [17,18].

Remarkably, disintegrins, isolated from snake venoms bind integrins with high affinity and can durably block their interaction with ECM [19]. Therefore, in cancer treatment., venom disintegrins [3,20,21,22,23] offer potential therapeutic alternatives to anti–tumour chemotherapeutic agents or may act in synergy with them as anti–angiogenic agents. By example, venom of king cobra Ophiophagus hannah was assessed to present strong anti–angiogenic effects in in vivo assays in yolk sac of zebrafish embryos [24]. Anti–angiogenic activity of Contortrostatin, a disintegrin from *Agkistrodon Contortrix* snake venom was evaluated through inhibition of migration, invasion and altered Matrigel–induced tube formation by human umbilical vein endothelial cells without affecting cell viability [25].

In our research of some new natural antagonists of neoangiogenesis from snake venoms, we found that the venom of *Daboia mauritanica* (*Dm*) contains at least one very active inhibitor of the endothelial cell functions involved in new vessel formation. We detail, hereinabove, isolation of this active inhibitor from the crude venom and its partial identification by sequencing and mass spectrometry. We describe our investigation about its effect on proliferation, adhesion, migration and in vitro tubulogenesis model of human dermal microvascular endothelium cells (HMECs) as well as their antiadhesive effect on the integrin ligands themselves. We elucidated the cellular targets of this inhibitor through various adhesion assays.

## 2. Results

### 2.1. Active Peptide Isolation Managed by Anti–proliferative Activity on HMECs

**Figure 1:**
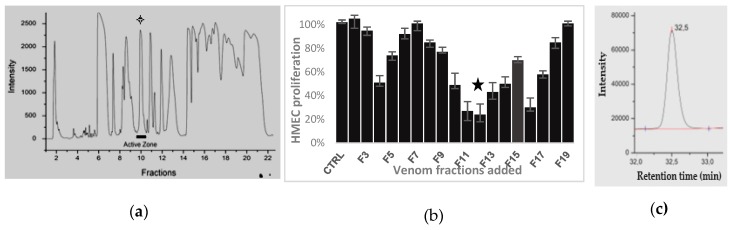
Isolation of active peptide: (**a**) Dm venom Chromatogram obtained after preparative HPLC in a C18 column, eluting 4 mL/min with a gradient of acetonitrile in 0.1% TFA in water (star points collection of fractions F11 & F12 of interest) (**b**) Effect on 96 h—HMEC proliferation (3 assays) of 5 µL amount of each chromatographic fractions (1m L) from 50 mg of crude venom obtained via 3 min–collection and 20–fold diluted. Star points effects of the most active fraction. (**c**) Analytic HPLC chromatogram of fraction F27,3 obtained in the (a) conditions but at a flow rate of 1 mL/min.

The venom HPLC profile by analytic HPLC showed a high number of components (Figure 1a). The *Dm* venom purification was done *via* ultrafiltration for to remove long peptides, followed by chromatography HPLC on reverse phase. After an ultrafiltration, the venom fractionation by 3 min, collections during 60 min chromatography through a reverse–phase HPLC C18 column afforded twenty fractions (called F1 to F20). They were submitted to a rapid test of modulation of 4 days proliferation of cells HMECs to investigate their antiproliferative activity. Screening of the venom fractions on HMEC proliferation allowed us to determine 3 domains of fractions with anti–proliferative activities on HMECs (Figure 1b). One active fraction was obtained at a short retention time (F4); two very efficient fractions F11 and F12 (star pointed in Figure 1a,b) were released later (27–32 min), before another one (F16) even more restrained. To isolate the peptides contained in F11 and F12, that may have anti–angiogenic properties, finer HLPC fractionations of the venom were done with collections on shorter periods (18 s. or 6 s.) on a C18 column, using the same gradient of eluting solvents. In HMEC proliferation assays, the strong inhibitor effects of 5 fractions apart 115 or 10 apart 110 from respectively the 18s– or 6s–collection were observed (results in Figure S1 of supplementary materials). Average masses of two main components in these active fractions, P_1_ (14,080 ± 1 Da) and **P_2_** (7721 ± 1 Da) were characterised by using mass spectrometry MALDI–ToF (matrix assisted laser desorption ionization–time of flight) or ESI (electrospray ionization) for a higher accuracy (spectra given in Figure S2 of supplementary materials).

As proved by mass spectrometry spectrum and HPLC analytical HPLC chromatogram (Figure 1c), the 6s–fractionation allowed us also to isolate peptide P_1_ in one of these fractions, fraction F27.3 released after 27.3 min of retention. This peptide was proved active against HMEC tubulogenesis as further showed. The second species P_2_ was isolated in two steps. A prefractionation by size exclusion on flash preparative FPLC was applied to the crude venom. It was followed by separation of products of one fraction *via* semipreparative HPLC on inverse phase in the same conditions that described above (chromatograms given in Figure S3 of supplementary materials). This second species was found inactive on proliferation and adhesion of HMEC (data not shown).

### 2.2. Identification of a P_1_ Moiety

**Figure 2:**
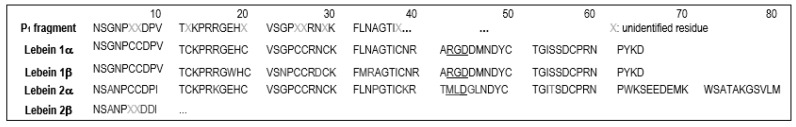
Sequence of peptide P_1_ compared to that of Lebein subunits.

A partial Edman sequencing of 37 N–terminal residues of peptide P_1,_ and a research inside database BLAST on Assembled Genomes [26], allowed us to find the close identity of P_1_ sequence with the N–terminal residues of a known disintegrin purified from *Macrovipera lebetina* venom, the alpha subunit of 64 residues of the heterodimer Lebein–1 (Figure 2). Attribution of the unidentified residues X to half–cystines is consistent with an alignment of the P_1_ structure with those of Lebein–1α. The subunit beta of this heterodimer has a slightly different sequence [27]. No peptide sequence close to this second subunit was identified from P_1_ sequencing. However, analysis of the active fractions by MALDI–ToF mass spectrometry showed that P_1_ has a very close average molecular weight to that of Lebein–1 determined from MALDI–ToF spectrometry measurement (mw: [M + 1H]^+^ = 14,083.4 Da) [27], within the error tolerances. As dimeric disintegrins formed by two subunits of approximatively 67 residues usually appear to contain four intrachain disulphides and two interchain cystine linkages [28], we estimated the Lebein–1 mass value from its sequence with 10 half–cystines [29]. Its calculated average (14,096.19 Da) and monoisotopic (14,086.84 Da) mass values, were slightly heavier than the founded mass values of P_1_ (monoisotopic mass =14,074 ± 1 Da from ESI mass spectroscopy). It is important to notice that, Lebein–1 and also another heterodimer disintegrin, named Lebein–2 [30], with a little higher mass (14,735 Da) from the *Macrovipera lebetina* venom, were supposed to be contained also in the *Dm* venom and were found to be agonists of laminin and able to target the β1 integrins. Lebein–1 was also recently proved to have anti–tumour properties against melanoma [31] and colon cancer [32].

### 2.3. Effect on HMEC Cell Viability and Concentration Dependent Proliferation

The P_1_ concentrations in fractions of interest obtained by several preparative HPLC and used in several tests were estimated indirectly by two modified Lowry methods [33]: either Biorad protein assays based on the method of Bradford [34] or either Pierce modified Lowry protein assay [35] using absorption of a dye complexe with BSA (bovine serum albumin) as reference. After P_1_ attribution to the Lebein–1 family, pure P_1_ fractions (1 µL) were quantified again *via* UV spectroscopy using spectrometer Nanovue at 280 nm assuming for the P_1_ molar extinction coefficient that of Lebein–1. The P_1_ concentrations in the active fractions were found 3.6–fold higher than the values estimated from Biorad test or close to that from Pierce assays. We concluded that a twenty–fold diluted sample of fraction F27.3 (225 µg/mL, 16.0 µM from UV spectroscopy measurements) containing 11.3 µg/mL (802.6 nM) of P_1_ was able to inhibit HMEC proliferation for more than 85%. P_1_ percentage in the crude lyophilized *Dm* venom was found lower than 0.83% after purification.

**Figure 3:**
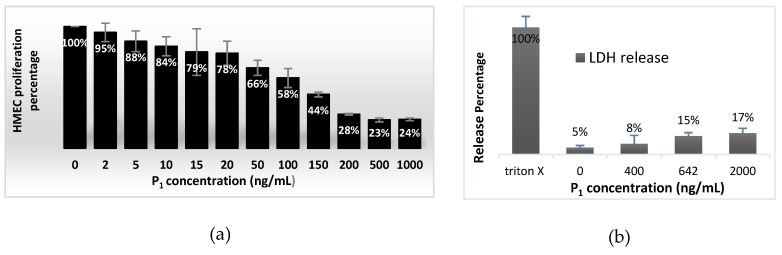
Effect of P_1_ on cell viability. (**a**) HMECs were incubated (2 assays) for 96h with various concentrations of P1 (in 100 µL per well), then with 0,5mg/mL MTT. After solubilisation with DMSO, the absorbance was measured at 600 nm. (**b**) HMECs were incubated (3 assays) for 5h with various concentration of P1 (50 µL per well) estimated through LHD release evaluated *via* Assay Kit TOX7.

As the toxicity of the P_1_ fraction in proliferation assays can be revealed by cell undocking from the well bottom after damages or death, we checked that the absorbance of the venom–treated wells, containing HMECs, was always superior to the absorbance of the starting cellular material. Therefore, from the first test results, P_1_ was observed to influence proliferation but not cell survival. Nevertheless, a P_1_ hypothetical cytotoxicity was checked more precisely by two accurate methods. In one test, the estimation of HMEC viability was measured after 4 days incubation of HMECs in presence of P_1_ fractions by estimating the adherent viable cells after staining mitochondria with MTT. The Formazan quantity formed in each well of the 96–well plate used corresponded to live cell number in each well. P_1_ affect 4 days proliferation of HMECs in a dose–dependent manner (Figure 3a). Viable cells gradually decreased with increasing concentration of P_1_. The IC_50_ value corresponding to reduction of HMEC viability was estimated equal to 89.89 ±23.54 ng/mL, from Hill equation [36] (with a hill coefficient of 1.21156), or equal to 96.98 ng/mL (6.9 nM) when inhibition was expressed in function of P_1_ logarithm (Inhibition = 0.1363 Ln[P_1_] – 0.1235). However, for a ten–fold concentration, the HMEC viability was still 24 ± 1% (Figure 3a). The absorbance measured at the highest P_1_ concentration tested (1 µg/mL) was close to the initial absorbance of the starting cellular material. An acute P_1_ cytotoxicity should have induce consequent cell damages followed by cell death and would have been characterized by a decrease in absorbance down to zero. Therefore, P_1_ amounts appeared not to affect survival of HMECs but inhibited greatly proliferation. In another test (Figure 3b), the possible cell membrane damages (lysis or loss of membrane integrity) were assessed measuring leakage of lactate dehydrogenase (LDH) into the medium. As enzyme LDH is known to reduce NAD^+^ present in medium into NADH, measurement of the LDH release was estimated through spectrophotometrically detection of a coloured Formazan derivative (soluble in water phase), issued from reaction of NADH with a tetrazolium dye added in the middle following Assay Kit TOX7 protocol. Results showed clearly that for 5h incubation of HMECs with a P_1_ concentration of 400 ng/mL (28.4 nM), necrotic lyses expressed by LDH release were a tiny process inducing only 3% of cell death, that was less than the natural death incidence. It increased to only 17% for a five–fold P_1_ concentration.

Both results suggest that the P_1_ effect on cell proliferation and formation of capillary–like structures is likely not due to necrotic death of HMECs induced by P_1_ cytotoxicity.

### 2.4. Effect on Angiogenesis

Angiogenesis induction is characterized by invasion of the basement membrane by vascular endothelial cells followed by their proliferation, and adhesion in sprout, then tandem migration, and maturation in capillary–like structures [10]. We thus tested the effect of P1 on various aspects of angiogenesis.

#### 2.4.1. HMEC Adhesion Assays

**Figure 4:**
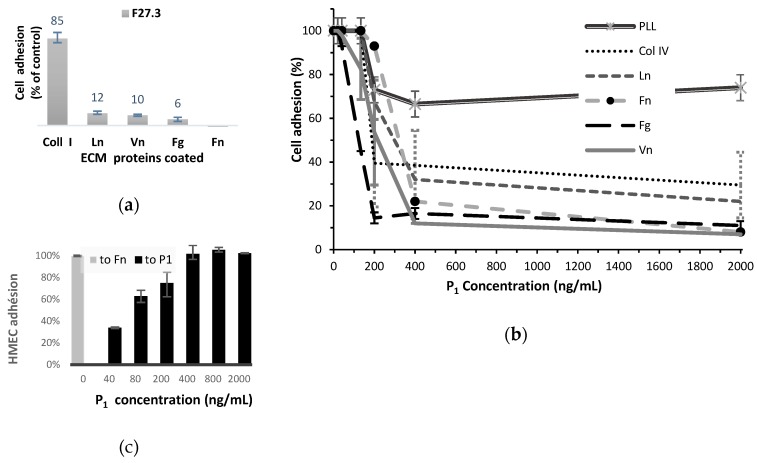
HMECs (25,000 in 50 µL) were allowed to attach (**a**) to various ECMs, in the presence of peptide P_1_ at concentration equal to 2 × 10^3^ ng/mL. (2 assays), (**b**) at various concentrations (6 assays); (**c**) to P_1_ used as a matrix at various concentrations, assuming adhesion to fibronectin (Fn) as a control (2 assays).

In the absence of appropriate ECM contacts, mainly mediated by integrins, endothelial cells undergo programmed cell death [37,38]. The networks of ECM laminin and type IV collagen (with the smaller protein nidogen and proteoglycans) form the molecular basis of the basement membrane. However, in the process of angiogenesis, the endothelial cells interact also with vitronectin, fibronectin, and fibrinogen. In order to investigate the P_1_ effects on the behaviour of endothelial cells, we first performed cell adhesion assays using a large array of purified ECM proteins, such as laminin (Ln), fibronectin (Fn), vitronectin (Vn), fibrinogen (Fg), collagen (Col) I or IV. Pre–treatment of HMEC with a relatively high dose of P_1_ (2 × 10^3^ ng/mL; 142 nM) from the venom fraction F27.3 greatly inhibited adhesion to ECM proteins, excepted to collagen I (Figure 4a). The inhibition of adhesion to fibrinogen, vitronectin, laminin and collagen IV was dose–dependent (Figure 4b). The values of IC_50_, as well as the maximal inhibition values are given in Table 1. The venom peptide inhibited peculiarly strongly the HMEC adhesion to fibrinogen (86% inhibition for a P_1_ concentration of 200 ng/mL or 14.2 nM). A twofold concentration allowed to inhibit in a similar extent the HMEC adhesion to vitronectin or fibronectin matrixes (88% and 80% inhibition respectively). The peptide was less efficient to inhibit HMEC adhesion to laminin I or collagen IV matrixes, with less than 70% inhibition at 29 nM P_1_.

To assess whether the effect of P_1_ on adhesion processes was integrin–dependent, we checked P1 effect on HMEC adhesion on Poly L–Lysine (PLL curve in Figure 4b). Adhesion to PLL was slightly inhibited (26%) in the P_1_ presence, traducing that a small part of the inhibition phenomenon of HMEC adhesion may occur outside the integrin recognition pathway.

In order to check whether P1 interacts with cell surface, we repeated the adhesion assay using the peptide as a matrix. As shown by Figure 4c, a consequent HMEC adhesion to P_1_ in 400 ng/mL (28.4 nM) concentration was observed in 1 h and it was comparable to that to fibronectin used as control.

#### 2.4.2. HMEC Migration Assays in Boyden Chamber

**Figure 5:**
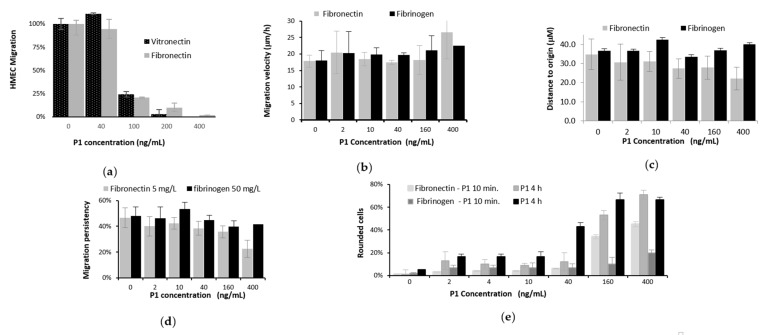
Dose–effect of peptide P_1_ on migration: (**a**) in a Boyden chamber of 30,000 HMECs per 100 µL– well when attracted during 5 h by ECM (2 assays) (**b**) checked by video–microscopy during 4 h of HMECs (10,000 /300 µL–well) after **P_1_** contact on 50 µg/mL fibrinogen or 5 µg/mL fibronectin ECM, measuring velocity, (**c**) distance to origin, (**d**) direction persistency, (**e**) cell shape (5 assays for b–e).

Inhibition of angiogenesis could be related to inhibition of the endothelial cell migration. Direction of EC migration is regulated by chemotactic, haptotactic, and mechanotactic stimuli [39]. To assess the potency of peptides to inhibit angiogenesis, HMEC migration assays in the presence of peptide P_1_ were performed in a Boyden chamber using vitronectin or fibronectin as attractant. Migration alteration by the venom peptide was dose–dependent (Figure 5a). A consequent inhibition of haptotaxy, was already observed for a P_1_ concentration of 100 ng/mL (7.1 nM) or either 200 ng/mL for fibronectin or vitronectin respectively as attractant.

#### 2.4.3. Video Microscopy of Locomotion of HMECs

In mesenchymal migration, the cytoskeletal remodelling, allowing the endothelial cells to extend, to contract, and to throw their rear toward the front and to progress forward, is due to activation of several signalling pathways [31]. This mesenchymal migration of HMECs in presence of various doses of the venom peptide was studied using video–microscopy in 24–well plate using fibronectin and fibrinogen as ECM. A subtle analyse of the migration parameters was done with Metamorph software. Fibrinogen (50 µg/mL) matrix, to which HMEC cell adhesion was unambiguously inhibited, was first used for the migration assays. HMEC migration characterized by measures of the travelled distance, distance to origin, velocity, and directional persistency values did not appear to be strongly altered in the presence of P_1_ during 4h (Figure 5b–d). However, deformation of cell shape appeared at P_1_ concentrations higher or equal to 160 ng/mL (11.4 nM), when P_1_ was incubated 4 h with the migrating cells (Figure 5e). The phenomenon of cell rounding observed was not surprising, as it could be attributed to a partial ungluing of cells from the extracellular matrix, to which cell adhesion was known to be strongly altered by P_1_. The P_1_ effect on migration on fibronectin showed a more tenuous inhibiting effect of cell adhesion. As it can be seen in Figure 5b–d for the 5 µg/mL matrix concentration, but also for two others tested (1 or 10 µg/mL, results not shown), distance or velocity of cell migration were not really perturbed. However, a similar cell rounding phenomenon was already observed when P_1_ was just added, and it was enhanced with the incubation time and with the P_1_ concentration. The data suggest that on both ECMs, HMEC cell migration changed gradually from mesenchymal to amoeboid migration with increasing amounts of P_1_ fraction. These modifications are already obvious at a P_1_ concentration of 160 ng/mL (11.4 nM).

#### 2.4.4. Tubulogenesis Assays

**Figure 6:**
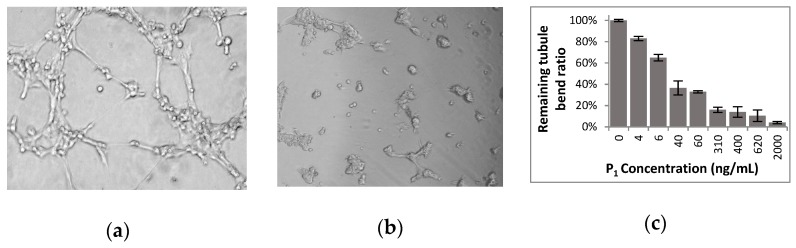
(**a**) Honeycomb structures formed in 6 h by HMECs (10,000 cells / 50 µL well) on Matrix BD Matrigel^TM^. (**b**) Cell aggregates formed in the same conditions, but in presence of 400 ng/mL peptide P_1_. (**c**) Estimation of the remaining tubule bends with Metamorph software *via* 5 photographs per well in presence of various P_1_ amounts (3 assays).

Tubulogenesis of the capillaries starts by an organization in loops of the endothelial cells that looks like honeycomb structures. This process was mimed in vitro by plating HMECs for 6h on a basement membrane matrix (BD Matrigel^TM^). In a preliminary study, amount of each venom fraction from the 18s– or 6s–collections containing peptide P_1_ visibly alter the formation of loops (Figure 6a) in extent that were qualitatively estimated (results in Table S1 of supplementary materials). Cell organization was secondly valued *via* the length sum of the linear cell packs and tubule bends formed. P_1_ at small concentrations altered the formation of organized structures mimicking the capillary tubulogenesis (see Figure 6a). The IC_50_ value was found smaller than 20 ng mL^−1^ (1.4 nM). Therefore, P_1_ appeared to be an anti–angiogenic peptide interesting to identify and to further test on the various steps of angiogenesis.

### 2.5. Identification Assays of Cell Targets of the Venom Peptide P_1_

The homology with Lebein–1 suggests that the venom peptide P_1_ may play role of a disintegrin. Our first results, detailed above, on inhibition of HMEC adhesion and migration, but also on impairment of tubulogenesis, corroborated this hypothesis. We expected first that, as found for Lebein–1 [19], P_1_ interacts with the laminin–binding β1 integrins such as α3β1and α6β1. Endothelial cells express various integrins for their proliferation, adhesion, migration, and differentiation, although they are differently activated and expressed in quiescent cells or in pathological angiogenesis. At least, seven integrin heterodimers are present in the endothelial cells: the collagen IV–binding integrin α1β1 [18], the laminin–binding integrin α6β1 [40,41,42], the joint collagen I / laminin–binding integrins α2β1and α3β1 [17], the fibronectin–binding integrin α5β1 [43], the joint fibronectin / vitronectin–binding integrins αvβ1and αvβ5 [14,41], and the joint fibronectin / vitronectin / fibrinogen–binding integrin αvβ3 [34,44,45], which is expressed mainly in angiogenesis. Preliminary studies in our laboratory about HMECs founded that these cells showed a strong expression of integrins αvβ3_,_ and some lower expression of αvβ5 (50% lower_,_), of α5β1and of the subunits β1, α3 and α6 [46]. These cells weakly expressed β1, αvβ8, α2β1, and do not express αvβ6and αIIb integrins.

#### 2.5.1. Tumour Cell Adhesion Assays in Presence of the Venom Peptide

**Figure 7:**
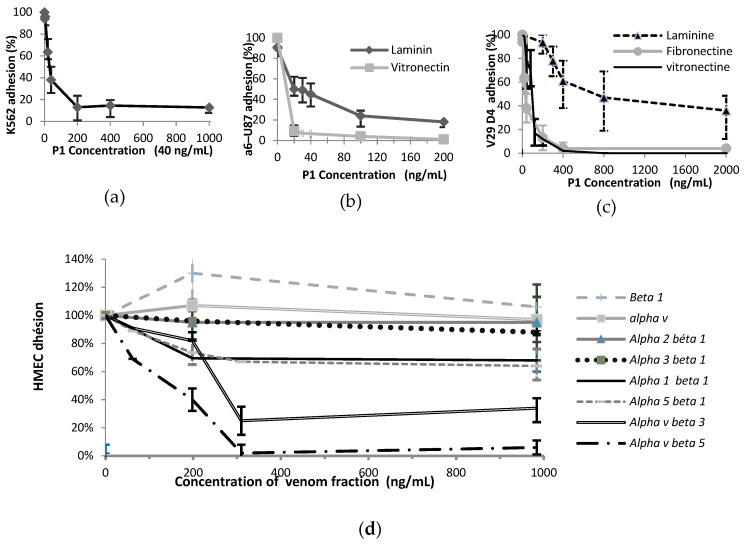
Effect of peptide P_1_ on adhesion (**a**) of K562 cells (50,000/well–2h – 3 assays) to fibronectin; (**b**) of α6–U87 cells (50,000/well– 30 min – 3 assays) to vitronectin et laminin; (**c**) of HT29D4 cells (50,000/ well – 2 h – 4 assays) to some various ECM; (**d**) modulation of HMECs (20,000 / well – 1 h – 4 assays) to various anti–integrins.

To identify the possible integrins targeted by peptide, we checked the P_1_ effect on adhesion of various tumour cell lines on a specific ECM protein, when it is known involving in each case mainly one integrin. We used the pairs (cells PC12 from pheochromocytoma of the rat adrenal medulla / type I collagen) [47] for α1β1, (erythroleukemia cells K562 / fibronectin) [48] for α5β1, (glioblastoma cell variants α6–U87 / laminin 1) [49] for α6β1, or (α6–U87 / vitronectin) for αvβ3 and αvβ5, (human colon adeno–carcinoma clonal cells HT29–D4 / laminin 1) for α6β4, (HT29–D4 / vitronectin) for αvβ5, (HT29–D4 / fibronectin) for αvβ6targeting. The results are detailed in Figure 7.

It was found that peptide P_1_ could not block the adhesive function of integrin α1β1 involved on the PC12 adhesion to collagen I, as the highest dose of P_1_ used (2 µg/mL) inhibited less than 20% adhesion of PC12 cells (data not shown). As demonstrated by Figure 7d, P_1_ was not efficient for blocking HT29 adhesion to laminin 1, involving integrin α6β4, since adhesion was still about 36% for the highest P_1_ concentration (2 µg/mL) and an IC_50_ ≈ 720 ng/mL (51.1 nM). In opposite way, P_1_ inhibited efficiently, at low concentrations (IC_50_ ≈ 30 ng/mL ≈ 2.1 nM), K562 adhesion to fibronectin *via* integrin α5β1. The inhibition was almost complete (87%) for a concentration of 200 ng/mL (14.2 nM) (Figure 7b). The P_1_ effect on U87–α6 cell adhesion to laminin 1 *via* integrin α6β1 was also effective for very small concentrations (IC_50_ ≈ 20 ng/mL ≈ 1.4 nM; Figure 7c). An 82% inhibition of adhesion was obtained for a 14.2 nM P_1_ concentration (200 ng/mL). P_1_ inhibited also efficiently U87–α6 adhesion to vitronectin (IC_50_ ≈ 10 ng/mL ≈ 0.7 nM; 100% inhibition for 14.2 nM P_1_ concentration), probably because of its effect on αvβ5 integrin. Indeed, a whole inhibition of HT29–D4 adhesion to vitronectin *via* the same αvβ5 integrin was obtained at 28.4 nM (IC_50_ ≈ 35 ng/mL ≈ 2.5 nM; Figure 7d). P_1_ displayed a great efficiency also in inhibiting the fibronectin / HT29–D4 interaction involving the αϖβ6 integrin (IC_50_ ≈ 2.5 nM; Figure 7d). Therefore, peptide P_1_ blocked in a dose–dependent manner various tumour cell attachment to ECM and could be an anti–integrin.

#### 2.5.2. Adhesion Assays to Anti–integrins in Presence of the Venom Peptide

To confirm the P_1_ anti–integrin behaviour, we inspected its interaction towards the various integrins involved in angiogenesis such as α1β1, α2β1, α3β1, α4β1, α5β1, α6β4, αvβ3 and αvβ5. HMECs treated with P_1_ were let to adhere to anti–integrin antibodies used as substratum. The results are given in Figure 6e. As expected by the above results, the venom peptide inhibited peculiarly strongly the HMEC adhesion on antibody anti–αvβ5 and anti–αvβ3. Maximal inhibitions (98% and 85% respectively) were obtained for a similar P_1_ concentration of 315 ng/mL. However, the IC_50_ value approximated 145 ng/mL in the first case and 245 ng/mL in the second. Inhibition of HMEC adhesion to antibodies anti–α5β1or anti–α1β1was less efficient and only limited to 30% for a concentration increasing from 200 ng up to 2 µg/mL of venom peptides. Peptide P_1_ could not inhibit HMEC adhesion to antibodies anti–α3β1or anti–α2β1, and to the antibodies to the subunits α6 or β1. Both last results were surprising because in contradiction with the precedent result on U87–α6 cell adhesion. Furthermore, treated HMECs were not homogenously spread on the antibody anti–α6 surface and are particularly agglomerated in the centre of the wells. This phenomenon induced a higher adhesion than 100% in comparison to the untreated cells.

## 3. Discussion

Peptide P_1_ isolated from *Daboia mauritanica* venom, which presents a strong similitude with disintegrin Lebein–1 isolated from *Macrovipera lebetina* venom, was proved to inhibit proliferation of HMECs with a maximal effect obtained for a concentration as low than 200 ng/mL (14.2 nM). The presence of peptide P_1_ also disrupted interactions of HMECs with the ECM proteins involved in angiogenic process. P_1_ was a strong inhibitor of the receptors of fibronectin, fibrinogen, vitronectin, collagen IV and laminin–1 on HMECs. A maximal inhibiting effect (70 to 90%) on 1h adhesion was encountered already for a P_1_ concentration of 400 ng/mL (28.4 nM), when it induced in 5h at the same concentration less than 3% of cell lysis. P_1_ perturbed also the mesenchymal migration of HMECs towards vitronectin and on fibronectin or fibrinogen at lower P_1_ concentrations (lower than 200 ng/mL–14.2 nM). Furthermore, P_1_ at 310 ng/mL (22 nM) concentration inhibited more than 80% of formation of the honeycomb organization of HMECs on Matrigel, a mimic of the tubulogenesis process.

In the angiogenesis process, multimeric vitronectin was shown regulating VEGF–induced angiogenesis, as it was found to bind VEGF and enhances VEGF–induced VEGFR–2/Src activation in endothelial cells (*via* effects on αvβ3 receptor) [50]. These cells will change in tip cells and will direct the nascent vessel. Moreover, the formation of the capillary sprout requires degradation of basement membrane and formation of an interim extracellular membrane from interstitial ECM proteins vitronectin, fibronectin, and fibrinogen. To this new ECM will adhere and migrate the stalk endothelial cells behind the tip one. After the meeting with another tip cell and fusion, there is perfusion, oxygenation, and maturation of the new vessel with deposit of a new basement membrane involving collagen IV and laminin–1 where phalanx endothelial cells adhere, migrate, and enter in quiescence after anchorage, helped by a fibronectin network. Our results in vitro on the ECM proteins show that P_1_ could act at the different steps of the in vitro anti–angiogenic process.

Lebein 1 is reported blocking α4β1, β5β1, α6β1and α7β1integrins [26,28]. Integrin α7β1is found on the surface of skeletal myoblasts and myofibers and is involved in their linkage to the ECM and the maintenance of the tension within tissue [51]. Consequently, inhibition of this integrin leads to progressive muscle dystrophy and myotoxic effect that were observed for *Macrovipera* venom. And indeed, in cell attachment assays, Lebein–1 inhibited myoblast attachment not only to laminin, but also to fibronectin. Lebein–1 was said not showing a specific, divalent cation–dependent interaction with the collagen–binding integrins α2β1 or α1β1 [30]. We obtained information on P_1_ from HMEC adhesion assays to antibody anti–integrins, although competition against anti–α_IIb_β_3_ and anti–α7β1 were not checked, as these integrins were not involved in angiogenesis. The interactions of P_1_ with integrins α5β1 and α1β1 were demonstrated, as the peptide competed with and won against the HMEC bindings to the corresponding anti–integrins antibodies. As P_1._ targets α5β1 integrin ligand, P_1._ could inhibit the K562 cell adhesion to fibronectin (IC_50_ ≈ 30 ng/mL ≈ 2.1 nM). A similar result was found in a study on Lebein–1 [30].

Contrarily, P_1._ failed to target integrins α2β1or α3β1. This result is coherent with the facts that P_1_ could not inhibit adhesion of PC12 cells to collagen I (via *α*1β1) and reduced only partially endothelial cells adhesion to collagen IV, which is mediated through α2β1and α1β1. We failed to prove by tests on anti–integrins, its recognition of integrin α6β1, as P_1_ was not efficient to inhibit HMEC adhesion to anti–α6 or anti–β1 subunit. However, we remarked in adhesion tests on anti–α6, an agglomeration of HMECs treated with P_1_ in the centre of the wells (results not shown), that could suggest a partial ungluing of HMECs from antibody anti–integrin surface, followed by a gluing between suspended or still adherent cells. This phenomenon could be explained if P_1_ is a dimer like Lebein–1 presenting two disintegrin patterns, one by each monomer, that can link two cells *via* integrins. Moreover, the inhibition by P_1_ of HT29–D4 cells adhesion on laminin 1 is in favour of its α6β1recognition. Besides, P_1_ blocked α6–transfected U87 cells adhesion to laminin 1 involving α6β1and partially inhibited HMEC adhesion on the same ECM which involves α6β1 but also α3β1integrin. P_1_ recognition of α6β4, could not be directly proved either. However, P_1_ partially inhibited HT29–D4 adhesion to laminin 1 mediated by this receptor.

P_1_ was shown also to block some α_V_ integrins and particularly αvβ3, αvβ5, and αvβ6. Direct evidence was given by peculiarly strong inhibitions of the HMEC adhesion on antibodies anti–αvβ3 or anti–αvβ5. P_1_ interaction with αvβ6integrin was coherent with inhibition of HT29–D4 adhesion to fibronectin. P_1_ interactions with αvβ3, α_V_β5 can explain that P_1_ is a strong inhibitor of receptors of fibrinogen (αvβ3), vitronectin (αvβ3, αvβ5) and fibronectin (αvβ3, αvβ5 and α5β1) of HMECs in adhesion assays [52]. As αv integrins regulate integrin–linked kinase (ILK) and then cell proliferation [53], partial impairment of cell attachment due to the presence of P_1_ could indeed also affect cell proliferation.

It is worth noting that the two monomers of disintegrin Lebein–1, as ECMs vitronectin, fibronectin, fibrinogen and collagen, contain the Arg–Gly–Asp (RGD) sequence recognized by cell integrins. The P_1_ sequencing did not allowed us to discover the linkage patterns of P_1_ towards α_V_ and α_1_ integrins receptors. However, its inhibitory properties in adhesion let us think that P_1_ is a disintegrin and contains such linkage patterns. Indeed, several studies on various disintegrins issued from snake venom allow to correlate their structure to their ability to blocks specific integrins [54]. The RGD pattern blocks the β subunits of the β1 and β3 integrins (such as α8β1, α5β1, αvβ1, αvβ3 and αIIbβ3) [55], but it is because of others residues such as the amino acid adjacent to the active RGD motif on the C–terminus or extracellular divalent cations (such as Ca^2+^and Mg^2+^) that the α subunit is selectively recognized [56,57]. The sequences Trp–Gly–Asp (WGD), Val–Gly–Asp (VGD), and Met–Gly–Asp (MGD) have been reported to be potent inhibitors of integrin α5β1, but WGD also targets αvβ3and αIIbβ3, when Met–Leu–Asp (MLD) targets the α4β1, α4β7 and α9β1 integrins [58]. Owing the strong analogy of a partial P_1_ sequence with the Lebein–1 alpha monomer, and because of its ability to recognize the α5β1, αvβ3 and αvβ6integrins and to function as a glue between adherent and suspended cells, we suppose that P_1_ exhibit also two RGD–like sequences.

Our results are not in contradiction with the recently reported apoptotic and antimetastatic properties of Lebein–1 that was reported to a have an anti–angiogenic effect through inhibition of VEGF expression in colon tumour cell lines [32]. Using hepatocellular carcinoma cells (HepG2), injected in blood circulation, such as some representative model cancer cells colonizing liver, Rosnenow et al. [59] analysed in detail the Lebein–1 effect on liver metastasis in vivo. Lebein–1 interfered with integrins that mediate interactions of tumour cells with the perisinusoidal ECM in liver and inhibit tumour cell arrest and/or extravasation in vivo. Lebein–1 was found to efficiently and completely block, in a dose–dependent manner, HepG2 cell attachment to both laminin–1 (involving integrins α6β1 and α6β4) and fibronectin (involving integrins αvβ1, αvβ5, αvβ6, and in a lower extent α5β1 and αvβ3) [60]. The RGD sequences of Lebein–1 explained its inhibitory potential of cell–fibronectin interactions. However, the Lebein–1 capacity to inhibit α6β1or α6β4 integrin binding to laminin–1 was entirely RGD–independent. The authors failed to find the conditions of adhesion of HT29LMM cells to laminin 1. The *Daboia mauritanica* venom peptide P_1_ appeared to target with high affinity the matrix receptors αvβ5 and αϖβ6involved in HT29–D4 (or U87) adhesion to respectively fibronectin or vitronectin. It is worth noting that integrin αvβ6mediates the potential for colon cancer cells HT29 to colonize in and metastasize to the liver [61] Its over–expression promotes MMP–9 secretion, enhances migration on fibronectin, and the survival of cancer cells in the liver. The over–expression of β6–integrin protect HT–29 colon cancer cells from growth inhibition and apoptosis induced by the chemo drug 5–Fluorouracil (5–FU) used for the treatment of colon cancer [62]. These results between others indicate that β_6_–integrin may constitute a therapeutic target in colon cancer therapy but also in gastric carcinoma [63,64]. The Lebein–1–like disintegrin properties of P_1_ show that P_1_ has a great potential in these perspectives.

## 4. Conclusions

We concluded that peptide P_1_ isolated from snake *Daboia mauritanica* venom to be a disintegrin very close to Lebein 1 from *Macrovipera lebetina* venom. Owing these disintegrin properties, we named P_1_ as Dabmaurin–1.

From the cell adhesion experiments to extracellular matrix ECM, Dabmaurin–1 appeared to target with high affinity the matrix receptors α5β1, α6β1, αvβ3, αvβ6 and αvβ5 and less efficiently α6β4 and α2β1. From HMEC adhesion experiments to antibodies anti–integrins, the Dabmaurin–1 interactions with the integrins α1β1, α5β1, αvβ5, αvβ3 were demonstrated, when those with integrins α2β1 or α3β1, were found negligible. An imposing array of integrins is involved in the angiogenesis control, including α1β1, α2β1, α3β1, α4β1, α5β1, α6β4, αvβ3, αvβ5 [65,66]. Therefore, Dabmaurin–1 recognizes a broad panel of the integrins particularly involved in the angiogenesis control, which are α1β1, α4β1, α5β1, α6β4, αvβ3, αvβ5. However, Dabmaurin–1, as a Lebein–1–like molecule, may have a great potential as a therapeutic in colon cancer therapy, but also in gastric carcinoma *via* its anti–β6 integrin properties, as this β6–integrin is well expressed in such tumour development [60]. As the vitronectin receptors αvβ6and αvβ3 are involved in U87 cells, Dabmaurin–1 might present interesting properties to study in the struggle against glioblastoma.

## 5. Materials and Methods

### 5.1. Materials

Dulbecco’s modified Eagle’s medium (DMEM), RPMI 1640 medium and fetal bovine serum (FBS) were purchased from Thermofisher Scientific (Waltham (MA), USA) Penicillin and streptomycin were purchased from GIBCO (Cergy–Pontoise, France). BD Matrigel^TM^ and bovine serum albumin (BSA) were purchased from BD Bioscience (Paris, France). Hydrocortisonhemisuccinate, epidermal Growth factor (EGF), blue thiazoyltetrazolium bromide, human fibrinogen and laminin–1 and type IV collagen were from Sigma (Mannheim, Germany). Type I collagen was from Biosciences and human fibronectin from Chemicon (Temecula, CA, USA). Human vitronectin was purified according to Yatohgo et al. method [67].

Integrin monoclonal antibodies anti–αv (rat mAb 69.6.5) was produced in the laboratory [68]. Anti–integrin anti–α2β1 (clone Gi9) and anti–α5β1 (clone Sam–1) were purchased from Millipore. Anti–α3β1 (C3VLA3), anti–β1 (clone Lia1/2), and rat monoclonal anti–α6 (clone GoH3) were from Immunotech (Marseille, France). Mouse monoclonal anti–αvβ5 (clone P1F6) as well as anti–αϖβ (clone LM609) were purchased from Chemicon. Rabbit anti–rat immunoglobulins antibody IgG was purchased from Sigma. The optical microplate reader was a labsystems multiscan RC ascent apparatus 600 nm. The used microscope was a Nikon Eclipse TE2000–E (Solent Scientific, Portsmouth, UK) apparatus with a camera CCD.

The human cells HT29–D4 clone derived from colonic adenocarcinoma were obtained in our laboratory [69] and were routinely cultured in DMEM containing 10% FBS. The U87 (ATCC) variants α6–U87 were obtained by retroviral strategy [49] and were grown in EMEM supplemented with 10% FBS. Human leukemia cells K562 (ATCC CCL–243) were cultured in RPMI 1640 medium containing 10% FBS. PC–12 rat pheochromocytoma cells (ATCC CRL–1721) were maintained in RPMI 1640 medium supplemented with 10% horse serum and 5% FBS. HMECs (Human Microvascular Endothelial Cell line 1) were originally developed by Ades [70] and obtained from the Cell Culture Laboratory in the Hôpital de la Conception (Assistance Publique Hôpitaux de Marseille, Marseille, France) were cultured on gelatin–coated flasks in MCDB–131 medium containing L–glutamine (1%), penicillin–streptomycin (1%), hydrocortisone hemisuccinate (0.1%), EGF (0.1%) and FBS (10%). All cell lines were maintained at 37 °C in 5% CO_2_.

### 5.2. Separation and Analysis of the Venom Fractions

#### 5.2.1. Venom Source

Venom of *Daboia mauritanica* was extracted from alive adult snakes of the serpentarium of Latoxan Company. After extraction, venom was immediately lyophilized and stored at −20 °C until used.

#### 5.2.2. Separation

The *Dm* venom was first submitted to an ultrafiltration through a 12–14 KDa MWCO membrane filter. The venom filtrate was then fractionated by semi–preparative HPLC on reverse phase in a 1260 Infinity Binary HPLC apparatus from Agilent Technology, eluting at a flow rate of 4 mL/min using in 0.1% trifluoroacetic acid (TFA) in water (*v/v*), a 60–min linear gradient of 0% to 60% of the mixture (acetonitrile/water/TFA–90/10/0.1). Using a Chromolith^®^ SemiPrep RP–18e (MERCK) (100 × 10 mm) column, a collection every 3 min afforded twenty fractions (F1 to F20) from a filtrate of the venom (1 mL injected containing 7 mg lyophilized venom in pure water). Using a C18 Eurospher column (120 × 16 mm), the crude venom (1mL injected with 25 mg lyophilized venom in pure water) was also fractionated in 210 fractions of 20 s–collections. A more precise fractionation with 6 s collections was also applied at retention times between 26 and 28 min. The strongest active peptide P_1_ was separated by this way in a few quantities from another one, P_2_, that had close retention time. Product P_2_ was isolated also by semi–preparative HPLC on C18 Eurospher column in the same conditions on the 9th fraction obtained after size exclusion of the crude venom (75 mg/mL, 12 fractions) on flash–preparative FPLC using Sephacryl S–100 HR gel filtration and elution with acetate ammonium 0.1M pH 6.

The HPLC analyses for checking purity and homogeneity of the collected fractions were done by injection of the 20–200 µl samples in a Purospher^®^ STAR RP–18 endcapped from MERCK or a Nucleosil 300 –5C18 PPN column (250 × 4 mm), from Macherey Nagel, using an SHIMADZU Analytic HPLC apparatus and upper eluting conditions but at a 1 mL flow rate. The molecular contents of fractions were detected by UV spectrophotometry at 214 nm (for the peptide bonds) and 280 nm (for the aromatic group).

The collected fractions were lyophilized and dissolved in 100 µl of PBS before use in microquantities in biological tests. Storage of any aqueous or lyophilized fractions was done at −20 °C respectively.

#### 5.2.3. Analysis by Mass–Spectrometry

The isolated peptides P_1_ and P_2_ (10 pmoles in 0.7–1 μL of 69,7% H_2_O / 30% MeCN / 0.3%TFA) of active fractions were characterized by spectrometry MALDI–ToF using an α–cyanohydrocinnamic acid (HCCA) matrix (0.7–1 μL equal volumes of saturated solution at 10 μg/μL in MeCN or in 50% MeCN/ 0.3% TFA/H_2_O. Spectra recorded in linear positive method LP12 kDa (Bruker microflex) or HMASS 5–20 kDa were externally calibrated with suitable standards as Prot cal I of Brucker with a laser (λ = 337 nm, W = 75.2 µJ, 500 shots of υ = 10 Hz), and were analysed by a Bruker Daltonics flex analyzis software (Bruker, Palaiseau, France). The fractions were checked also in a reflectron mode but gave no additional data. 

Peptides were analysed by nano–electrospray LC–MS (Dionex Ultimate 3000, Thermofisher scientific, llkirch, France) coupled to a hybrid Q Orbitrap mass spectrometer equipped with a nano–ESI source (Q exactive ThermoFisher scientific, llkirch, France). The spectra were treated using Xcalibur Software (ThermoFisher Scientific, llkirch, France).

#### 5.2.4. N–terminal Edman Sequencing

*N*–terminal Edman degradation of the P_1_ peptide (100 pmoles in 1 µl) was performed using an automatic sequencer (Procise 494, Applied Biosystems, Thermofisher scientific, llkirch, France) associated with HPLC with an Applied PTH C18 column (220 × 2.1mm) and UV detector (269 nm) and using an Applied Biosystem analyser software (Model 610A 2.1) [71]. Sequence homology was evaluated by a computer search in the protein sequence database using BLAST program implemented in the protein–protein BLAST (blastp) search at http://www.ncbi.nlm.nih.gov.

#### 5.2.5. Quantification

The peptide concentration of the active fractions was estimated by the Biorad assay in a microtiter protocol using the brilliant blue G–250 Coomassie dye [29] or by the Pierce™ BCA Protein Assay (Thermofisher scientific, llkirch, France). For both tests, BSA was used as standard protein.

The concentrations of the pure fractions were estimated *via* UV spectroscopy (spectrometer Nanovue Plus from Biochrom of Havard Biosciences, Havard apparatus, les Ulis, France) at 280 nm, assuming for the P_1_ molar extinction coefficient that of Lebein–1 (ε_L_ = 12710 M^−1^ × cm^−1^) estimated by BLAST and ExPasy protparam tool [20,21,22,23] using the molecular weight (14088.8 Da) of Lebein 1 in a molecular form (C_565_H_471_N_189_O_190_S_23_) containing ten cystine bonds. Evaluation of the P_1_ maximum quantity ((8.28 µg) found by mg of the crude lyophilized *Dm* venom was estimated also *via* UV spectroscopy though the mass concentration of the active fractions pooled together. Concentrations of P_1_ in the active fractions were found higher than that found from Biorad test as peptidyl compositional differences between BSA and P_1_ cause variability in sequence coloration by the Coomassie dye which binds only to primarily basic amino–acid residues (such as arginine) and, to the aromatic ones. In contrary, the universal peptide backbone (and residues cysteine, cystine, tyrosine, and tryptophan), contributing in Pierce test to colour formation, helped to minimize variability in coloration between P_1_ and BSA.

### 5.3. Cell Proliferation Assays and Viability Measures with HMEC

#### 5.3.1. Cell Proliferation

HMECs (2500 /well) in 100 μL of culture medium were plated into two 96–well culture plates Falcon. and incubated 2h at 37 °C for cell to recover and adhere in a humidified atmosphere of 5% CO_2_. One plate was treated immediately for spectrophotometry revelation contained 3 wells with cells to serve as reference for optic absorbance of the starting cellular material (T_o_) and 3 wells with just PBS for a blank test (BT_1_). The wells of the second plate received in triplicate various venom peptide amounts (2 to 10 µl in PBS), excepted six of them that received only 5 µl PBS amount to serve as a second blank test (BT_2_) and a proliferation test of untreated cells. The cells in the plate were let to proliferate for 96 h at 37 °C in a humidified atmosphere of 5% (*v/v*) CO_2_. After removing the supernatant, adherent cells, washed with PBS, were fixed for 10 min with 1% glutaraldehyde in PBS, washed twice with water, stained in 30 min with 50 μL of 0.1% crystal violet and lysed with 1% SDS before absorbance reading (600 nm). The effect of the venom fractions on HMEC proliferation was expressed *via* the proliferation percentage, using the formula:

*Proliferation percentage* = *100% × (A of well − Aof* T_o_ − *A of* BT_1_) / *(A of untreated growth control − A of To − A of* BT_2_). (1)

The toxicity of the venom fraction can be revealed by an absorbance *A* of venom–treated wells inferior to that of T_o_.

#### 5.3.2. Measure of Viable Cells

HMEC (2500 cells/well) plated in 96–well plate in 100 μL of medium were incubated overnight at 37 °C for cell to recover and adhere, before adding venom fractions or PBS for control. After 96 h incubation, 10 μL of solution of blue thiazoyltetrazolium bromide (5mg/mL) in PBS was added for 1 h at 37 °C. Afterwards the supernatant was carefully removed, and each well was washed with PBS and received 100 μL DMSO (dimethyl sulfoxide) to solubilize the formazan derivative. The well absorbency A measured at 600 nm was used for viability calculation. The untreated wells by the venom peptide represented 100% viability. The effect of the fraction venom on viability of human cell lines panel was expressed via the viability percentage, using the formula: *Viability percentage = 100% × (A of treated cells /A of control cells)*. Cell proliferation was also checked.

#### 5.3.3. Cytotoxicity Assays

The cytotoxicity of venom fractions was determined by measuring the release of lactate dehydrogenase (LDH) activity into the medium. HMEC (10,000 cells/well) plated in a 96–well plate in 100 μL of culture medium were incubated 5h at 37 °C in the presence of venom fractions (or PBS for control). Total release of LDH (100% toxicity) was obtained by treatment of cells with 0.1% Triton X100 in incubation medium. The supernatants were collected, clarified by centrifugation 5 min at 600 g and 80 µL were submitted to LDH–based cytotoxicity kit (Sigma).

### 5.4. Tubulogenesis Assays

Briefly, Matrigel^TM^ (40 μL) from Engelbreth–Holm–Swarm Mouse sarcoma (BD, 10.2 mg/mL) were added to 96–well plates and allow to solidify for 30 min at 37 °C. HMEC in culture medium (10, 000 cells per well in 50 μL) were then added to Matrigel^TM^ in the presence or the absence of peptide for 5 h at 37 °C to form capillary–like structures. Capillary–like structures images were recorded using a Retiga 1300 camera (Qlmaging, Surrey, BC, Canada) and quantified by measuring the thread length of the capillaries.

### 5.5. Cell Migration Assays

#### 5.5.1. Cell Migration in Modified Boyden Chamber

In vitro cell migration assays (haptotaxis) were performed in modified Boyden chambers (NeuroProbe Inc., Bethesda, MD, USA) with porous membranes pre–coated with 10 μg/mL of fibronectin or vitronectin for 5 h at 37 °C as previously described [72].

#### 5.5.2. Videomicroscopy

Briefly, 24–well plates were coated 2 h at 37 °C with 10 μg/mL fibronectin or to 50 μg/mL fibrinogen. Cells (10,000 cells in 300 µL culture medium) and were let to adhere for 1h before washing with culture medium. Venom peptide samples were added to the wells and the plate was placed in microscope enclosure at 37 °C in 5% CO_2_ atmosphere. The migration was followed using a Nikon microscope taking 24 photographs in 4 h of three or four fields randomly chosen by well. Software Metamorph was used to analyse the migration parameters.

### 5.6. Cell Adhesion Assays

#### 5.6.1. Cell Adhesion Assay to ECM

Adhesion assays to ECM were performed as previously described [73]. Briefly Maxisorb 96–well plates were coated 2h at 37 °C or 12 h at 4 °C with purified extracellular matrix (ECM) proteins 10 up to 50 μg/mL in PBS and saturated with PBS/0.5% BSA. Cells in adhesion buffer (DMEM containing 1,2 g/L of Na_2_CO_3_, 15 mM HEPES at PH 7,3 and 0,2%BSA) were firstly incubated 30 min. at 20 °C with the active peptide P_1_ before being added to the wells (50,000 cells in 50 µL) and allowed to adhere to the substrata for 30 min (α6–U87 cells), 1 h (PC12 cells and HMEC) or 2 h (HT29–D4 and K562 cells) at 37 °C. Adhesion of K562 cells was performed in the presence of phorbol 12–myristate 13–acetate and MnCl_2_ in order to activate α5β1 integrin [50] and to promote high levels of ligand binding [51]. Afterwards, cells were fixed with 1% glutaraldehyde, stained with 0.1% crystal violet and lysed with 1% SDS. Absorbance A was then measured at 600 nm.

#### 5.6.2. Cell Adhesion Assay to Anti–integrin Antibodies

Maxisorb 96–well plates were first coated with 50 μL of rabbit anti–rat IgG (50 μg/mL in PBS), overnight at 4 °C. Wells were washed once with PBS and 50 μL of anti–integrin blocking antibodies (10 μg/mL) were added for incubation 2 h at 37 °C. Wells were then saturated with a solution of PBS/0.5% BSA. After washing, 50,000 cells in adhesion buffer in the presence or absence of the venom peptide were added to wells and allowed to adhere for 1 h 30 at 37 °C. Cell adhesion was measured as above.

#### 5.6.3. Cell Adhesion Assay to Peptide P_1_

Maxisorb 96–well plates were coated with 0.1 μL of peptide **P_1_** for 2 h at 37 °C and washed with PBS. After blocking with PBS/0,5% BSA, cells in adhesion buffer were treated or not with the appropriate blocking antibodies (5 μg/mL, 30 min) and then added to the venom peptide–coated wells. Cells were let to adhere 2 h at 37 °C and then quantified as described above.

## Figures and Tables

**Table 1:** IC_50_ and maximal inhibition of HMEC adhesion by P1 to various ECMs ^1^.

ECM	IC_50_ of P_1_	Inhibition Maxima ^2^
Col IV	189.2 ± 1.4 ng/mL (~13.5 nM)	29 ± 6%
Ln	205.4 ± 8.9 ng/mL (~15.2 nM)	22 ± 1%
Fn	307.1 ± 2.0 ng/mL (~21.9 nM)	8 ± 1%
Fg	127.6 ± 8.1 ng/mL (~9.6 nM)	11 ± 2%
Vn	200.8 ± 2.1 ng/mL (~14.4 nM)	7 ± 1%

^1^ from Hill equation [25]; ^2^ [P_1_] = 2 × 10^3^ ng/mL.

## Supplementary Materials

The following are available online at https://www.mdpi.com/2072-6651/12/2/102/s1, Figure S1: HMEC–proliferation assays (in 96 h) in presence of chromatographic fraction amounts from *Dm* venom (50 mg), Figure S2: Characterization of the active *Dm* venom fractions from the chromatographic 6s–collection, Figure S3: Purification and characterisation of peptide P_2_, Table S1: Effect of 2.5μL venom fractions on the structure organization of HMEC (10,000 cells / 50 μL well) in 6 h on BD Matrigel^TM^.
