# Supplementary material for: Isolation of an Anti–tumour Disintegrin: Dabmaurin–1, a Peptide Lebein–1–like, from Daboia mauritanica Venom
Source: Toxins (Basel). 2020 Feb 5;12(2):102. doi: 10.3390/toxins12020102 (PMC7076848; doi:10.3390/toxins12020102)
Supplement: Supplementary file 1 [file toxins-12-00102-s001.pdf]

Supplementary materials: Isolation of an Antitumour Disintegrin: Dabmaurin–1, a Peptide Lebein–1–like, from Daboia mauritanica Venom

Florence Chalier, Laura Mugnier, Marion Tarbe, Soioulata Aboudou, Claude Villard, Hervé Kovacic, Didier Gigmes, Pascal Mansuelle, Harold de Pomyers, José Luis and Kamel Mabrouk

1. **Isolation and characterisation of the active fractions from the *Daboia mauritanica* venom**

The 18s–collection of fractions from a 60 min–chromatography through a reverse–phase HPLC C18 column, of the *Dm* venom, led to 5 fractions apart 115, inhibiting HMEC proliferation, as shown in Figure S1a. They were labelled Fti–tj where (ti–tj) expresses a release period in minutes out of the column. Their retention times were spaced between 26.7 and 27.6 min in the eluting conditions. Collection on 6 s periods led to nine active fractions active against HMEC proliferation (Figure S1b).

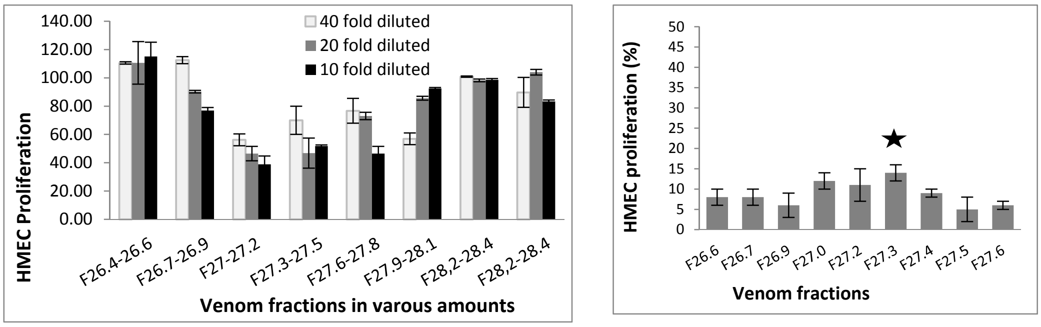

(**a**) (**b**)

Figure S1. HMEC proliferation in 96 h in presence of chromatographic fraction amounts from *Dm* venom. 3 assays done with a new venom sample (50 mg each) using lyophilized fractions in PBX (200 µL) **(a)** from 18s–collection and variously diluted **(b)** from 6s–collection and 20–fold diluted.

These 6s–collection fractions were characterised by their mass spectra using MALDI –ToF (Figure S2a and b and c) and/or electrospray ionization (Figure S2d). Two products P_1 and P_2 were characterised on a Microflex II Bruker MALDI–ToF mass spectrometer (Figure S2a and S2b). The analysis gave for P_1 : m/z (%) = 14080 ±13 $[(M+H^+)/1]$ – 7038 ±7 $[(M+2H)^{2+}/2]$– 4692 ±3 $[(M+3H)^{3+}/3]$. Compound P_1 which inhibited HMEC's proliferation was found particularly purified in one fraction (F27.3, star pointed in Figure S1c) with an average mass about 14080 Da (Figure S2c). The ESI mass spectrometry of one of these fractions containing P_1 and P_2 (Figure S2d to S2e) confirmed us that P_1 has an averaged mass of 14080± 1 Da calculated from the $[M+10H]^{10+}$ or $[M+11H]^{11+}$ ion pattern (m =10 ×1408.9998 – (10 × 1.00739) Da ≈ 14079.9 Da, or m ≈ 14078.9 Da respectively). Its isotopic mass was estimated at 14074.75 Da from the smallest line value (1280.53) of the spectrum pattern of the $[M+11H]^{11+}$ ion.

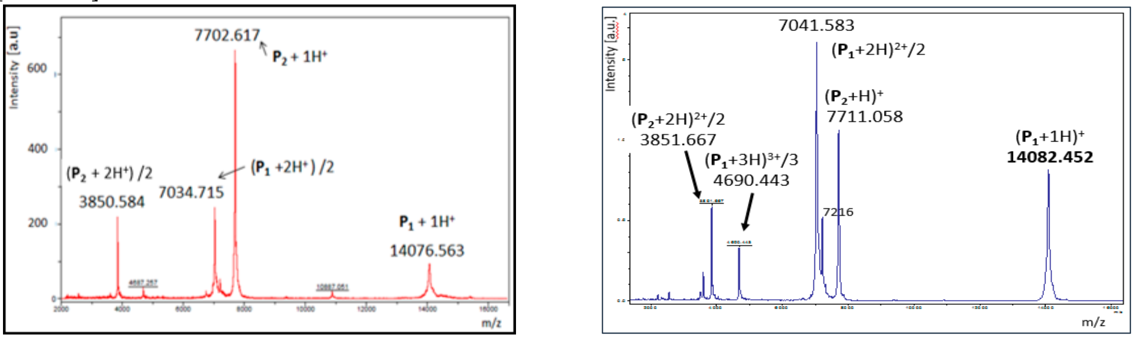

(**a**) (**b**)

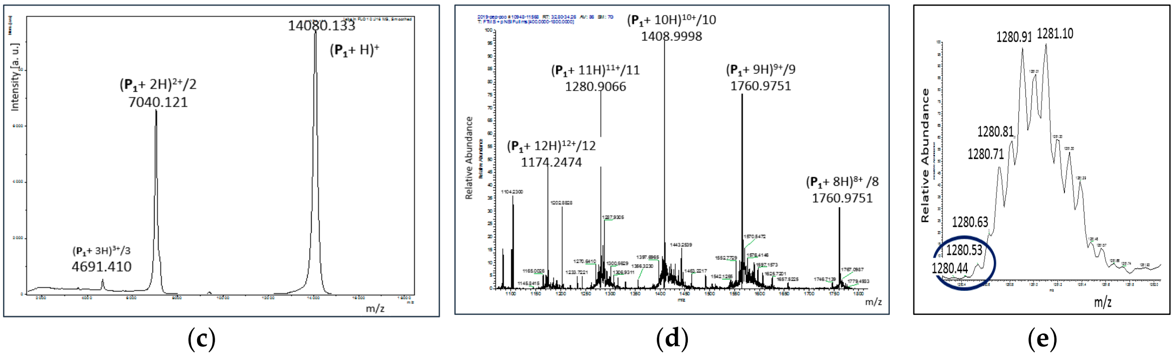

Figure S2. Characterisation of the active *Dm* venom fractions from the chromatographic 6s–collection : (**a**) MALDI ToF mass spectrum of F27.0, (**b**) of F27.2, (**c**) of F27.3; (**d**) ESI mass spectrum of F27.2 by focusing on P_1 signal (**e**) by focusing on the $[P_1+11H]^{11+}$ ion pattern.

The second species P_2 of the active fraction of 6s–collection chromatography was isolated in two steps. A prefractionation by size exclusion on flash preparative FPLC applied to the crude venom afforded the fraction F_{P2}, which was fractionated *via* a semi–preparative HPLC on inverse phase. The taller peak corresponded to product P_2. The corresponding chromatograms and MALDI–ToF spectrum (M/z: Mm+1H and M+2H/2) were given in Figure S3.

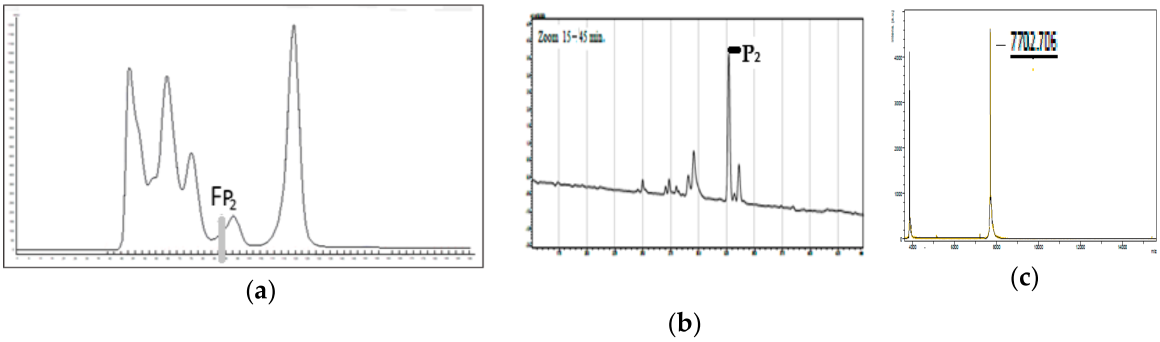

Figure S3. Purification and characterisation of peptide P_2**.** (a) Gel filtration chromatogram of *Dm* venom (Sephacryl S100, ammonium acetate 0.1M - pH6.8, flow rate of 1ml/min); (b) Chromatogram obtained after preparative HPLC using a C18 column, flow rate of 3 ml/min with a gradient of acetonitrile in 0.1% TFA in water; (c) MALDI–ToF mass spectrum of compound P_2.

2. Visual determination of tubulogenesis of capillaries –like structure formation

The effects of the HPLC fractions from the 18s– or 6s–collections containing peptide P_1 on the organization in 6h of the endothelial cells plated on basement membrane matrix (BD Matrigel™) was visually determinated and gave the results given (Table S1). Loops mimeing honey comb structures, semi–loops and cell packs were differentiated on the photographies obtained on the wells where HMECs were let to organise themselves on Matrigel in presence or not of the venom fractions. The fractions from the 18s–collection, F26.7–26.9 up to F27.9–28.1 altered unambiguously the loop formation. The fractions from the 6s–collection, F26.6 up to F27.6 altered strongly this loop formation.

Table S1. HMECs structural organization (10 000 cells / 50 μL well) in 6 h on BD Matrigel™

**Cell Organization**	**Honeycomb Structures----------------------**			**Cell packs----------------------**	
Shape on surface well	On whole surface	On partial surface	Semicircular	Linear	Dot shaped, Isolated cells
Corresponding photography	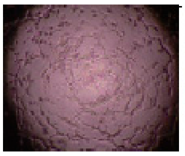	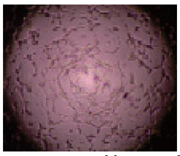	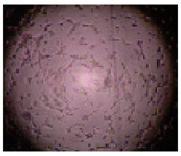	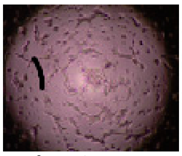	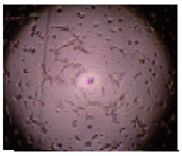
Effect of 2,5 μL	Effect of 2,5 μL amount of Dm *fractions*				
Amount *fractions* of 18s.–collection	F26.0–26.2<br>F28.5–28.7	F26.3–26.5<br>F28.2–28.4	F26.7–26.9<br>F27. 9–28.1	F27.0–27.2<br>F27.6–27.8	F27.3–27.5
of 6s–Collection	F26.0		F26.3	F26.6 ; F26.8<br>F26.9 ;<br>F27.5 ; F27.6	F27.0; F27.1;<br>F27.2; F27.3<br>F27.4;
